# Disparities in the increases of cervical cancer incidence rates: observations from a city-wide population-based study

**DOI:** 10.1186/s12885-022-09531-2

**Published:** 2022-04-15

**Authors:** Ke Li, Huan Xu, Suixiang Wang, Pengzhe Qin, Boheng Liang

**Affiliations:** 1 Department of Prevention and Control of Chronic Noncommunicable Diseases, Guangzhou Center for Disease Control and Prevention, No.1 Qide Road, Guangzhou, 510440 China; 2 Institute of Public Health, Guangzhou Medical University and Guangzhou Center for Disease Control and Prevention, Guangzhou, China

**Keywords:** Cervical cancer, Incidence, Time trend, Population-based study, Geographical disparities

## Abstract

**Background:**

Globally cervical cancer incidence rate has been declining continuously. However, an unfavorable trend has been observed in China during the past decades, and the underlying reasons remain unclear. We hereby explore the recent trends of cervical cancer incidence, as well as the underlying determinants using data from Guangzhou, one of biggest cities in China.

**Methods:**

City-wide cancer registration data were obtained from the Guangzhou Center for Disease Prevention and Control from 2004 to 2018. We used the Joinpoint regression models to estimate the average annual percentage change (AAPC) of age-standardized and age-specific incidence rates by regions and by histological subtype. Age-period-cohort models were applied to analyze the period and birth cohort effects on the time trends.

**Results:**

The age-standardized rates (ASRs) of cervical cancer incidence increased at an annual rate of 2.1% [95% confidence interval (CI): 1.0%-3.2%] during 2004–2018. The largest increase in ASRs was found for rural regions, with AAPC of 6.6% [95% CI: 3.7%-9.5%], followed by the suburbs (2.2% [95% CI: 1.0%-3.4%]), while there was no statistically significant increase in urban regions. The ASRs of adenocarcinoma increased faster than those of the squamous cell carcinoma (AAPC = 6.53% [95% CI: 5.0%-8.1%] versus 1.79% [95% CI: 0.8%-2.8%]). A downward trend in urban regions was found in the 20–49 age group, whereas an upward trend was found in the 50 + age group, especially in rural regions. An inverted V-shape was found for cohort effects, with the peak varied by regions, i.e., peaked in the 1966 and 1971 birth cohort in the urban and suburb regions, respectively. Period effects kept increasing during the study period.

**Conclusions:**

We systematically examine the disparities in the increases of cervical cancer incidence rates using city-wide data from Guangzhou. Extensive efforts are warranted to address the large urban–rural disparities in cervical cancer prevention. The combined strategies of vaccination, screening, and health education should be reinforced and locally customized.

## Background

Cervical cancer was the fourth most frequently diagnosed cancer and the fourth leading cause of cancer death in women, with an estimated 604,000 new cases and 342,000 deaths worldwide in 2020 [1]. Significant differences in cervical cancer incidence between developed and less developed countries were observed. About 84% of cervical cancer cases and 88% deaths occurred in low-resource countries [2]. Cervical cancer mortality rate has fallen over the past decades in many high-resource countries following the well-organized long-term screening programs for cervical cancer. However, stable or even rising trends in mortality rate have been observed in countries where mass screening activity is not practice, or conduced in low-quality or low-coverage [3].

In China, both cervical cancer incidence and mortality rates appeared to be on the rise [4, 5]. A recent study anyalzed cervical cancer data in two cities in different provinces showed that the urban–rural disparity in age-standardized incidence rate was greater than that between developed and less developed countries [6]. In addition, previous studies of different ethnic populations including Chinese on trends of cervical cancer incidence showed that the increase in incidence was mainly attributable to the 40–69 age group [4, 7–9]. In Guangzhou, the capital city of Guangdong province, China, due to the continuous increase in cervical cancer incidence since 2000 [10], a free screening program was launched in rural residents covered in suburban and county districts in 2009 [11].Whether this screening program reduced the urban–rural disparities in cervical cancer mortality remains unclear.

Hence, we systematically analyzed the differences among different age groups, regions and histological subtype using Jointpoint Regression using data from Guangzhou cancer registry. Our study aims at providing general information about distribution of cervical cancer in a big city of China before and after the launch of the screening program. The experience and evidence should be useful for other low- and middle-income countries.

## Methods

### Data source

Incidence data of cervical cancer were obtained from the Guangzhou Cancer Registry, which was launched in 1998 and has covered all districts in Guangzhou since 2004. All cancer cases were requested to be reported by a qualified hospital through a Network Direct Report System. For each incident cancer case, basic information and disease-related information was registered. Basic information includes registered identification number (ID), medical ID, Identity Card Number, name, sex, birth date, occupation, ethnicity, resident permanent address, phone number. Disease-related information includes cancer site, International Classification of Disease (ICD) 10^th^ edition code, methods for diagnosis, treatment, prognosis and pathological report if available, date of diagnosis, name of hospital and doctor, and International Classification of Disease for Oncology, Third Edition (ICD-O-3). Since 2010, all cases were referred to the Community Health Service Centers for regular follow-up. Quality control was conducted by general practitioners in the Community Health Service Centers, who double checked all information to ensure the accuracy. For residents who had treatment outside Guangzhou, information was obtained from the National and Guangdong Province Cancer Registry. Cervical cancer cases were identified using ICD-10 codes C53 [12]. Morphology was classified based on ICD-O-3: squamous cell carcinoma (SCC) (8050–8084), adenocarcinoma (AC) (8140–8550/8570–8576), and not otherwise specified (NOS).

**Fig. 1:**
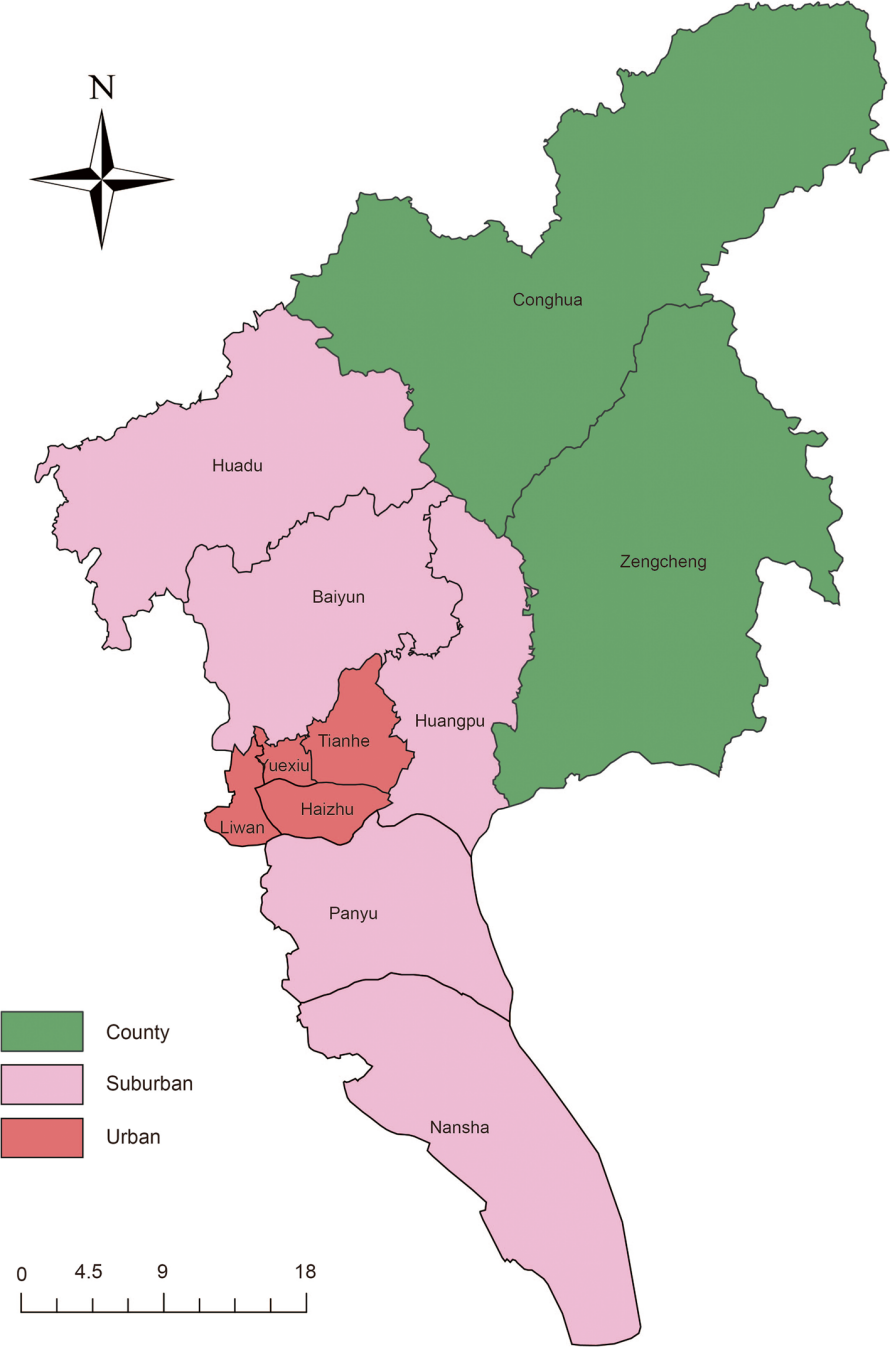
Map of urban, suburban and county regions in Guangzhou

Annual population data were from the Guangzhou Municipal Statistics Bureau (NBS). Based on the municipal government development report [13], four developed districts (Yuexiu, Haizhu, Liwan and Tianhe) without rural residents were categorized as urban region, five medium developed districts (Baiyun, Panyu, Huangpu,Nansha and Huadu) with some rural residents as suburban region, and two less developed districts with major rural residents (Zengcheng and Conghua, located in the northeast of Guangzhou) as county region (Fig. 1).

### Statistical anlysis

We used Excel 2016 to construct the database and calculate crude incidence rates (CIRs), age-specific rates, age-standardized incidence rates (ASIRs), standard error and 95% confidence interval (CI). ASIRs were calculated by the direct method using the world Segi’s population [14]. Joinpoint Regression Program was used to analyze trends Version 4.0.4 [15].

We used age-period-cohort (APC) model to estimate the incidence of cervical cancer and to examine the sex-specific effects of age, period and cohort on incidence using a web tool for age-period-cohort analysis (https://analysistools.cancer.gov/apc/) [16]. The APC model assumes that the number of cervical cancer cases follows a Poisson distribution and that incidence rates are a multiplicative function of the included model parameters, making the logarithm of the rates an additive function of the parameters. The APCs for each age group, called “local drifts”, can be generated from log-linear regressions. The web tool was also used to estimate the overall annual percentage changes (net drifts) in age-standardized rates, which incorporate linear components of both cohort and period effects. The central age group, period and birth cohort was defined as reference group. In case of an even number of categories, the reference value was set as the lower of the two central values. Data were categorized into 12 age groups from 20–25 to 80–84 years in the APC model, together with 3 diagnosis period groups (2004–2008, 2009–2013, 2014–2018) and 14 birth cohort groups (1922–1926 to 1987–1991).

## Results

### Descriptive summary

Table 1 shows the characteristics of the incident cases in Guangzhou. From 2004 to 2018, the total number of cervical cancer cases was 8140. The mean (SD) and median age was 50.74 (12.18) and 50.0 years, respectively. Both mean and median age increased with time period. The 35–49 age group in 2004–2013 and 50–64 age group in 2014–2018 had the largest number of cases. Most cases had a histological type of SCC. From 2004–2008, to 2009–2013 then to2014-2018, the proportion of AC increased, whereas the proportion of SCC decreased.

**Table 1:** Incidence cases of cervical cancer in Guangzhou, 2004–2018

Characteristics	2004–2008		2009–2013		2014–2018		Total	
	N	%	N	%	N	%	N	%
Basis of diagnosis								
Histology	2027	92.14	2477	92.150	3010	92.56	7514	92.31
Death Certificate Only	10	0.45	9	0.335	8	0.25	27	0.33
Endoscopy and Radiology	64	2.91	81	3.013	80	2.46	225	2.76
History and Physical exam	44	2.00	45	1.674	63	1.94	152	1.87
Surgery and Autopsy (no history)	8	0.36	17	0.632	11	0.34	36	0.44
Other	47	2.14	59	2.195	80	2.46	186	2.29
Mean(SD) = 50.74(12.18)	47.10(12.02)		50.42(11.37)		53.48(12.27)		50.74(12.18)	
Median (IQ) = 50(42:58)	45(39:53)		49(42:57)		53(45–61)		50(42–58)	
District								
Urban	875	43.15	1075	40.02	1254	38.60	3204	40.24
Suburban	846	41.72	1103	41.06	1330	40.94	3279	41.18
County	307	15.14	508	18.91	665	20.47	1480	18.59
Age group								
20–34	265	12.05	149	5.54	173	5.32	587	7.21
35–49	1134	51.55	1244	46.28	1093	33.61	3471	42.64
50–64	599	27.23	982	36.53	1413	43.39	2994	36.78
65–74	122	5.55	221	8.22	385	11.81	728	8.94
75 +	80	3.64	92	3.42	188	5.78	360	4.42
Histological type								
Squamous cell carcinoma	1620	79.92	1945	72.359	2333	71.74	5898	78.49
Adenocarcinoma	231	11.40	345	12.835	466	14.33	1042	13.87
Unspecified and other	176	8.68	187	6.957	211	6.49	574	7.64

*Abbreviations*: *SD* Standard Deviation, *IQ* Inter Quartile

### Time trends of age-standardized incidence rates by region

**Fig. 2:**
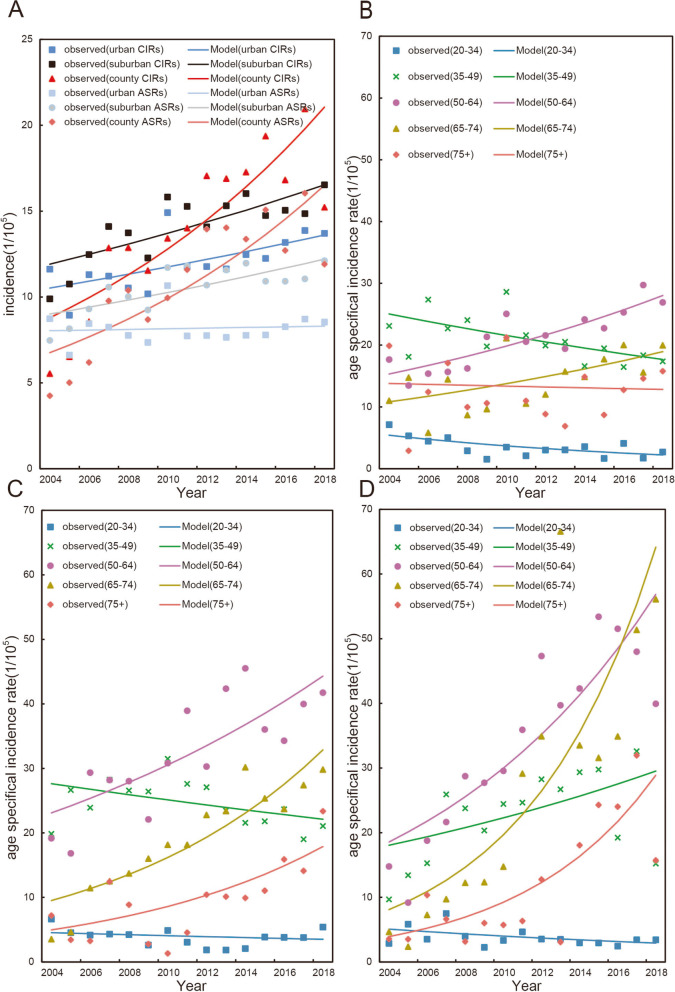
Trends of incidence rates for cervical cancer in Guangzhou, 2004–2018. **A** Trends of crude incidence rates (CIRs) and age standardized incidence rates (ASIRs) for cervical cancer in urban, suburban and county regions in Guangzhou; **B**, **C**, **D** Age-specific incidence rates for cervical cancer by year of diagnosis in urban, suburban and county regions in Guangzhou, respectively. Model was the fitting rate of the Jointpoint regression

Figure 2A, Tables 2 and 3 show significant increases in both CIRs and ASIRs for all regions and three regions of Guangzhou except the ASIRs in urban regions. For CIRs, we found an overall significant increase in average annual percent change (AAPC) of incidence rate in all regions (2.9%, 95% CI: 1.9%–4.0%, *P* < 0.05). The AAPC in urban, suburban and county regions was 1.9% (95% CI: 0.5%–3.2%), 2.4% (95% CI: 1.2%–3.6%) and 6.4% (95% CI: 3.8%–9.1%). After adjusting for age structure, ASIRs showed the same patterns with CIRs except urban regions. The AAPC of ASIRs in all regions of Guangzhou was 2.1% (95% CI: 1.0%–3.2%, *P* < 0.05), increasing from urban, to suburban and country regions (0.2% (95% CI: -1.2%–1.7%), 2.2% (95% CI: 1.0%–3.4%) and 6.6% (95% CI: 3.7%–9.5%), respectively).

**Table 2:** Crude incidences and ASRs of cervical cancer incidence between 2004 and 2018 in Guangzhou

District	Variable	2004–2008	2009–2013	2014–2018	total
Urban	N	875	1075	1254	3204
	population	8,163,786	8,922,596	9,569,772	26,656,154
	Crude incidence(1/10^5^)	10.72	12.05	13.1	12.02
	ASR(1/10^5^)	7.97	8.2	8.21	8.24
Suburban	N	846	1103	1330	3279
	population	6,916,470	7,571,546	8,605,099	23,093,115
	Crude incidence(1/10^5^)	12.23	14.57	15.46	18.05
	ASR(1/10^5^)	9.27	14.61	17.91	14.09
County	N	307	508	665	1480
	population	3,311,598	3,476,037	3,713,314	10,500,949
	Crude incidence(1/10^5^)	9.27	14.61	17.91	14.09
	ASR(1/10^5^)	7.2	11.52	13.85	10.97
ALL	N	2200	2688	3252	8140
	population	18,391,854	19,970,179	21,888,185	60,250,218
	Crude incidence(1/10^5^)	11.96	13.46	14.86	13.51
	ASR(1/10^5^)	8.99	9.74	10.19	9.84

**Table 3:** Temporal trends in CIRs and ASIRs for cervical cancer in Guangzhou, 2004–2018

Rate	Trend 1		Trend 2		AAPC 2004–2018 (95%CI) (%)
	Year	APC (95%CI) (%)	Year	APC (95%CI) (%)	
CIRs					
All regions	2004–2018	2.9*(1.9,4.0)			2.9*(1.9,4.0)
Urban regions	2004–2018	1.9*(0.5,3.2)			1.9*(0.5,3.2)
Suburban regions	2004–2007	12.3(-1.2,27.6)	2007–2018	1.3(-0.1,2.7)	2.4*(1.2,3.6)
County regions	2004–2007	34.4(-0.3,81.3)	2007–2018	4.20*(1.5,7.0)	6.4*(3.8,9.1)
ASRs					
All regions	2004–2018	2.1*(1.0,3.2)			2.1*(1.0,3.2)
Urban regions	2004–2018	0.2(-1.2,1.7)			0.2(-1.2,1.7)
Suburban regions	2004–2007	12.2(-2.1,28.4)	2007–2018	1.1(-0.4,2.6)	2.2*(1.0,3.4)
County regions	2004–2012	13.4*(6.3,20.9)	2012–2018	-0.3(-7.0,6.9)	6.6*(3.7, 9.5)

*AAPC* Average annual percentage change

^*^annual change is significantly different from zero (*P* < 0.05)

### Time trends of age-specific incidence rates by region and by histological subtype

The AAPC of age-specific incidence rate is shown in the Table 4, Figs. 2B ~ D and 3. The incidence rates in the 20–34 age group showed significantly decreasing, especially in the urban regions (AAPC = -6.14%, *P* < 0.05). For the 34–49 age group, no statistically significant changes in the suburban and county regions were observed. The incidence rates in groups aged 50 + years were increased significantly over time. The AAPC in the county regions was greater than the suburban and urban regions in the groups aged 50 + years. The AAPC of women aged 65 + years in the county regions were more than 15% (*P* < 0.05).

**Table 4:** Temporal trends in age-specific cervical cancer in different regions of Guangzhou, 2004–2018

Age	AAPC(%, 95%CI)			
	All regions	Urban	Suburban	County
20–34	-3.91*(-6.7,-1.0)	-6.14*(-9.9,-2.2)	-1.83(-5.7,2.2)	-3.86(-7.6,0.1)
35–49	-1.04(-2.6,0.6)	-2.46*(-4.3,-0.6)	-1.57(-3.3,0.2)	3.57(-0.2,7.5)
50–64	5.08*(3.8,6.4)	4.40*(2.8,6.0)	4.76*(2.4,7.2)	8.33*(5.1,11.6)
65–74	8.93*(6.8,11.1)	4.08*(0.8,7.5)	9.26*(6.0,12.6)	15.92*(8.7,23.6)
75 +	5.42*(1.8,9.2)	-0.52(-5.0,4.2)	9.58*(3.8,15.7)	15.31*(8.4,22.6)

*Abbreviations*: *AAPC* Average annual percentage change, *95%CI* 95% confidence interval

^*^annual change is significantly different from zero (*P* < 0.05)

**Fig. 3:**
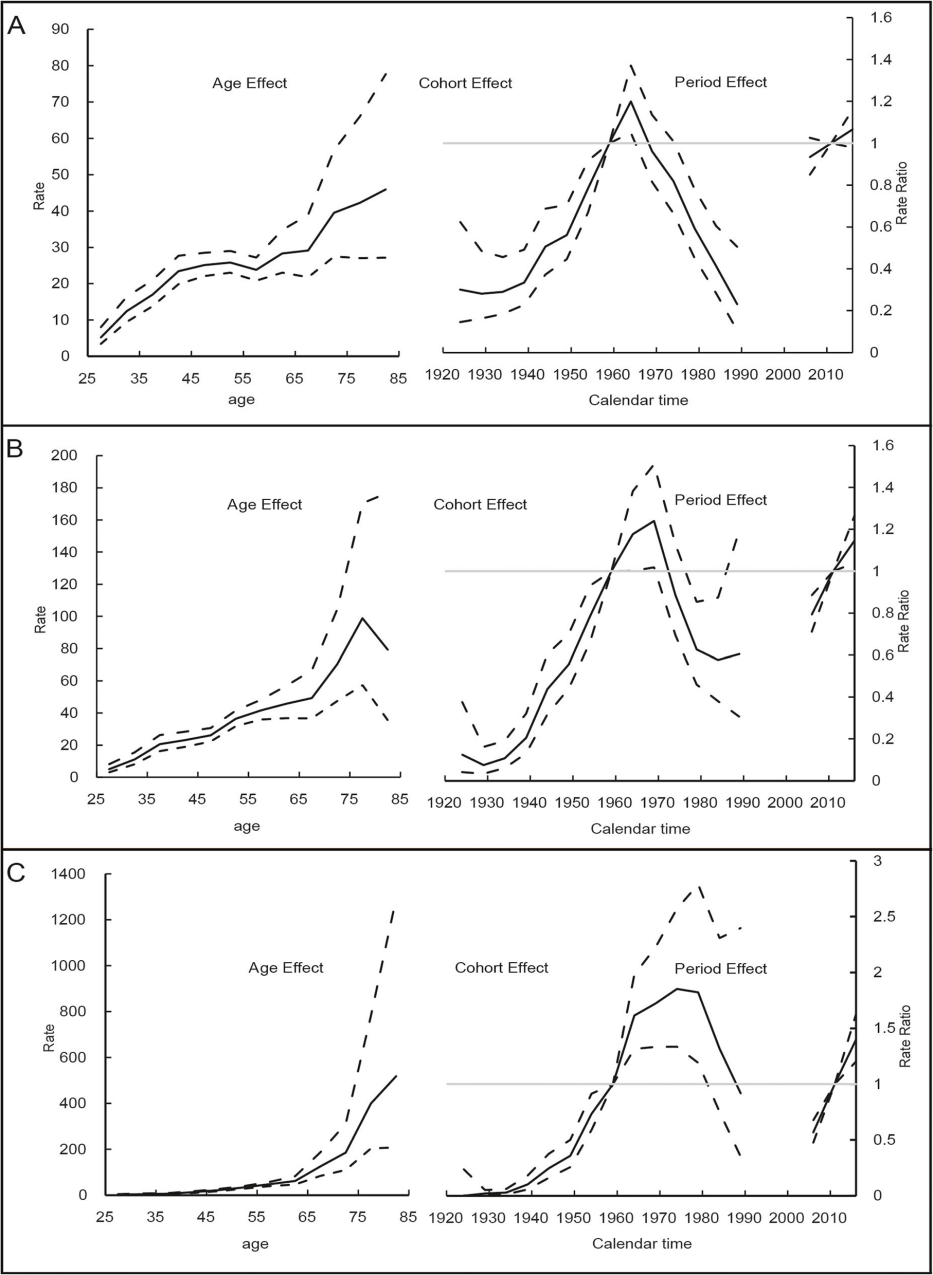
Age group-specific annual percent change (local drift) for incidence of cervical cancer for aged 20–84 years in Guangzhou, 2004–2018 in urban (**A**), suburban (**B**) and county (**C**) regions. The dotted lines represented their 95% confidence interval. The incidence rates showed significantly decreasing in the urban regions for the 20–34 age groups and increasing for the 50–74 age groups, especially for the county regions

Table 5 shows the ASIRs for the histological type of SCC increased in suburban regions and county regions. The increase was most pronounced in county regions, followed by the suburban regions (AAPC = 7.16% and 1.69%, respectively, both *P* < 0.05). For the histological type of AC, the ASIRs were increased by 5.70% in urban regions, 6.18% in suburban regions and 9.63% in county regions.

**Table 5:** Temporal trends in different histology of age-standardized incident rate for cervical cancer in Guangzhou, 2004–2018

Histological type	AAPC(%)			
	All regions	Urban	Suburban	County
SCC	1.79*(0.8,2.8)	-0.27(-1.4,0.9)	1.69*(0.3,3.1)	7.16*(4.7,9.7)
AC	6.53*(5.0,8.1)	5.70*(4.0,7.4)	6.18*(3.7,8.7)	9.63*(4.6,14.9)
Other	-0.57(-3.5,2.5)	-2.40(-6.9,2.4)	0.58(-3.1,4.4)	-0.02(-5.5,5.7)

*AAPC* Average annual percentage change, *AC* Adenocarcinoma, *SCC* Squamous cell carcinoma

^*^annual change is significantly different from zero (*P* < 0.05)

### Age-period-cohort analysis

**Fig. 4:**
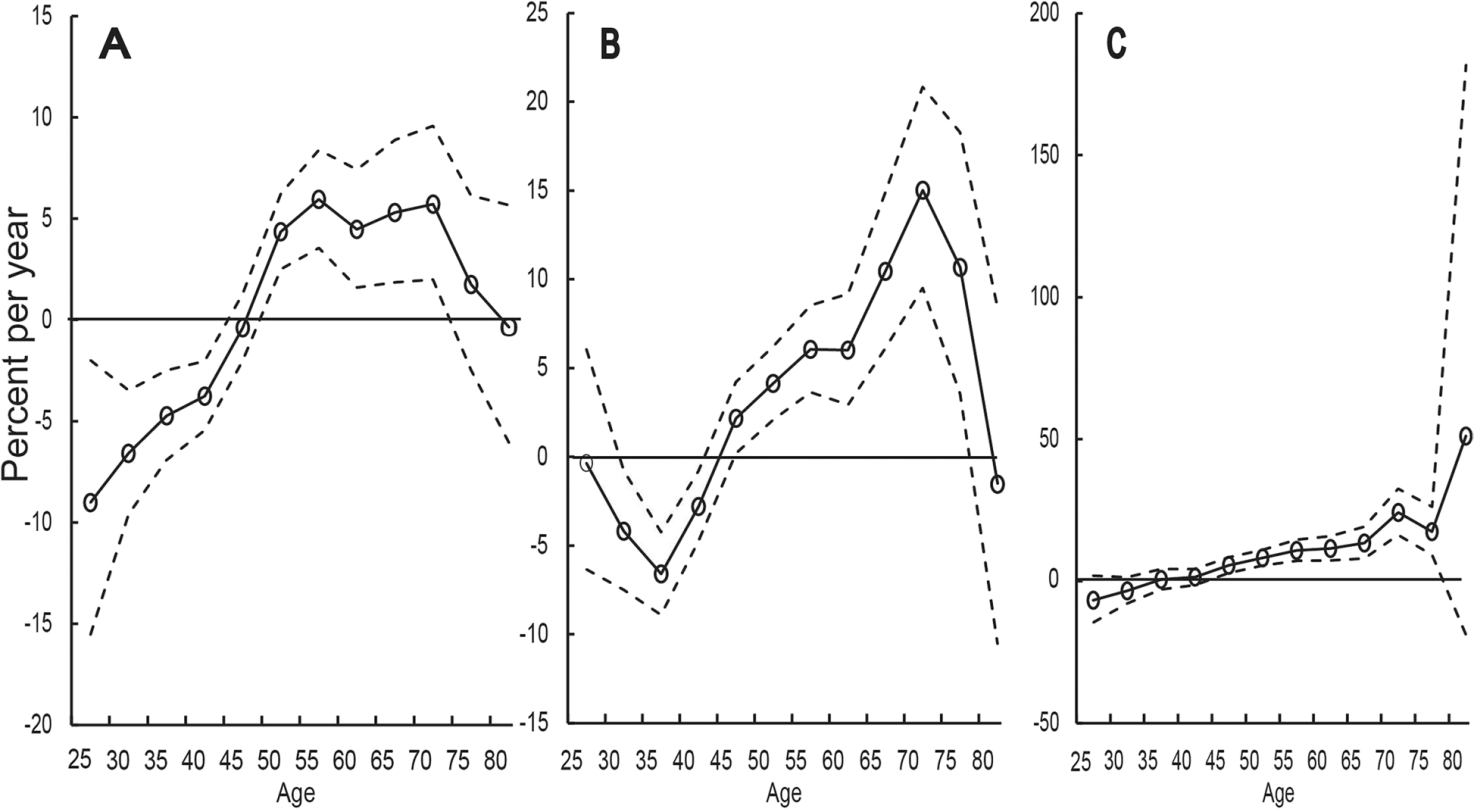
Estimated age-period-cohort effects for incidence of cervical cancer for aged 20–84 years in Guangzhou, 2004–2018 in urban (**A**), suburban (**B**) and county (**C**) regions. On the x-axis, 5-year age groups, birth-cohorts and calendar periods are defined by the first year of the interval. Reference cohort is 1957–1961.The age effects were longitudinal age curve. The dotted lines represented their 95% confidence interval

Figure 4 depicts the results of age-period-cohort model analysis. The period effects for the incidence of cervical cancer increased from 2004 to 2018 in suburban and country regions. In all regions, the rate ratio for cervical cancer incidence increased in those born between 1922 and 1966 after adjusting for age and period effects. Increasing trends were still observed in those born during 1966–1971 in suburban regions and those born during 1966–1981 in county regions. However, the risk ratio for cervical cancer incidence were decreased in women born after 1987.

## Discussion

During the past decades, incidence rates of cervical cancer appear to be increasing in Guangzhou. However, the incidence rates of cervical cancer have fallen in some high-income settings, including north America [8], northern and western Europe [17], and Australia and New Zealand, and South Korea [18], which were probably the result of successful cytological screening [19]. Our study showed increasing trends of cervical cancer incidence in Guangzhou from 2004 to 2018, which were driven by cohort and period effects. Similar increasing trends were also observed in other regions of China [4], Russia [20], some countries of Central and Eastern Europe [21] and Africa [7, 22, 23]. As high-risk human papillomavirus (HPV) types are necessary cause of cervical cancer, the increasing cervical cancer incidence in Guangzhou could be due to the following reasons. The first reason is the growing prevalence of high-risk HPV, which was high to 20.2% in female population during 2008–2017 [24], and the other might be due to screening, due to the knowledge gaps, attitudes of fear or embarrassment and the role of contacts and service models [25].

Although urban regions had the highest incidence rate of cervical cancer in Guangzhou in 2004, the increase in incidence was more sharply in county regions during past decades. The incidence in county regions has exceeded that in urban and suburban regions since 2010, and the similar patterns were also reported in Shanghai [26]. The crude incidence in urban regions were increased but showed no change after age-standardization, indicating that the increase was mainly due to the change in age structure (i.e., population ageing). The incidence showed a downward trend in the age groups of 20–49 years, and an increasing trend in the age groups of 50–74 years, which were consistent with the increasing risk in successive birth-cohorts born between 1922 and 1966s and decreasing risk after 1966s [27]. Unfortunately, no period effects were evident to support the effect of opportunistic urban-wide screening in the urban regions. In Hong Kong, despite well-organized population-based screening of cervical cancer has never been introduced, the observed decrease in incidence could be attributable to an increase in opportunistic screening from 1972 to 2006 [28, 29].

The crude and standardized rate of cervical cancer incidence increased in suburban and county regions, which increased mainly in the age group of 50 + years. The increase was more sharply in county regions than suburban regions. The APC analysis in our study showed that period effects increased from 2004 to 2018 in suburban and county regions, which was consistent with the implication of the local screening program. In Guangzhou, the local government has enormous investment in cervical cancer screening since 2009, and such program was main launched in major of county regions and some of the suburban regions. The screening was organized by the government and offered to voluntary rural women aged 35 to 64. The screened women first went to the primary screening hospital for cervical exfoliation smear and other tests. If there was abnormal, they were recommended to the District Maternal and Child Health Hospital for diagnosis. More than 600,000 women had been screened [11]. The period effect might be explained by the following reasons. This project may not have enough coverage, and were only conducted at one time point without follow-up. The birth cohort between 1922 and 1966 showed the same increasing trend in different regions, but the age group of the birth cohort decreased later in suburban and county regions than urban regions. The increasing cohort effects on the cervical cancer incidence rate in those born during 1922–1966 could be due to the more open sexual behavior after the reform and opening up after the 1980s and the lack of opportunistic screening conditions. With the improvement in health awareness and the increase in opportunistic screening, especially the occupational physical examination of many companies began to include cervical cancer screening in routine physical examination, the cohort effects on the incidence cervical rate has become decreasing since 1970s. The peak of the cohort effects varies across regions, which may be due to urban–rural inequity in medical resource allocation and health awareness in urban–rural regions.

Besides the incidence increase of SCC subtype that mimicked the overall trend, a rapid rise in the incidence of AC subtype was observed. An increase in adenocarcinoma incidence rates has also been observed in other countries like the United States [8] and Western Europe [9], even the SCC incidence rate has decreased. The sensitivity of Pap testing is higher for SCC and its precursors compared to AC [30]. The rates of SCC may have increased in line with AC if the screening not been widely available or disseminated [31]. SCC did not increase as much as AC in Guangzhou, which may be due to this reason.

Our analysis shows that opportunistic screening and the local government’s efforts in screening in rural regions have a partial effect. Greater efforts are needed to curb the continued rise of cervical cancer. A survey showed that only 20% of the women interviewed reported having ever had a Pap test and even less of those who received continuous screening. Also, women in rural regions were less likely to take a Pap test than those in urban regions [32]. The World Health Organization (WHO) recommends that all women between the ages of 30 and 49 years should be screened for cervical cancer at least once [33]. The American Cancer Society (ACS) recommends that individuals with a cervix initiate cervical cancer screening at age 25 years and undergo primary human papillomavirus (HPV) testing every 5 years through age 65 years (preferred); if primary HPV testing is not available, then individuals aged 25 to 65 years should be screened with contesting (HPV testing in combination with cytology) every 5 years or cytology alone every 3 years (acceptable). Individuals aged > 65 years who have no history of cervical intraepithelial neoplasia grade 2 or more, with severe disease within the past 25 years, and who have documented adequate negative prior screening in the prior 10 years, discontinue all cervical cancer screening [34]. Thus, based on our results and international experiences, we suggest that women aged 30 years or older should begin to be screened. Moreover, free Cervical Cancer Screening Program should be expanded to all regions of Guangzhou as a major public health benefit as early as possible in the face of continuously increasing incidence rate in older age groups. Systematic evaluation for the effectiveness of cervical cancer screening should be performed periodically and regularly.

There are several factors that might influence the participation in cervical cancer screening, including lack of knowledge and awareness of cervical cancer screening and its benefits, fear of pain and being diagnosed with cervical cancer, embarrassment, the lack of husband’s support for screening, and cultural factors [35, 36]. The cytological screening of cervical cancer requires high quality expertise and equipment. Rural county regions in Guangzhou often do not have a department of cytology or pathology. Individuals in rural regions need go to city hospital to screen. Therefore, a relevant health education policy should be developed, which aimed at raising women’s awareness on the risk factors of cervical cancer and the importance of early diagnosis. At the same time, the medical service capacity in rural regions should be enhanced and medical resources should be optimized.

Since the world’s first HPV vaccine became available in 2006, the authorities in China finally approved Cervarix (GlaxoSmithKline Biologicals SA, Rixensart, Belgium) in 2016, Gardasil 4 (Merck & Co, Inc, Kenilworth,NJ, USA) in 2017, and Gardasil 9 (Merck & Co, Inc) in 2018 in mainland China [37]. To expand vaccination, national level HPV vaccination strategy should be developed. Meanwhile, health education should be strengthened to raise public awareness on the efficacy of.

### HPV vaccine in order to increase vaccine uptake

Limitations of our study include lack of information on stage at diagnosis for most cancer cases and the hysterectomy data. Moreover, due to the lack of data, limited risk factors that might have affected cervical cancer incidence were examined. Hence, further studies are needed to explore factors explained the disparity of cohort and period effects between different regions in Guangzhou. Strengths of this study include our population-based analysis of the incidence trends for cervical cancer included high-quality and long-term surveillance data in Guangzhou, one of biggest cities in China. In addition, the observation period was relatively long (from 2004 to 2018) including the year the government started cervical cancer screening in suburban and urban regions. On account of the accurate and representative surveillance data, our results are convincing.

## Conclusions

In this city-wide analysis using data from Guangzhou, the cervical cancer incidence rates were increased during 2014–2018. The incidence rate in county regions has exceeded those of the suburban and urban regions since 2011 and the gap between county regions and suburban/urban regions has been gradually widened. Moreover, the incidence rate has increased fastest in county regions, followed by suburban regions. During 2004 and 2018, the 20–34 age groups showed a significant decrease, especially in the urban regions, whereas the 34–49 age groups in the suburban and county regions showed no significant changes and the 50 + age groups showed significant increase over time. We also found that the increase in AC incidence was more pronounced than the SCC incidence. Our results provide important evidence to facilitate further policy making, especially for establishing screening and HPV vaccination program for prevention and control of cervical cancer.

Extensive efforts are warranted to address the large urban–rural disparities in cervical cancer prevention. The combined strategies of vaccination, screening, and health education should be reinforced and locally customized.

## Data Availability

The datasets analyzed during the current study are not publicly available to protect the participants’ anonymity. But can be freely available from the corresponding author on reasonable request.

## Abbreviations

AAPC: Average annual percentage change
CIRs: Crude incidence rates
ASRs: Age-standardized rates
CI: Confidence interval
ICD: International Classification of Disease
ICD-O-3: International Classification of Disease for Oncology, Third Edition
SCC: Squamous cell carcinoma
AC: Adenocarcinoma
NBS: Municipal Statistics Bureau
APC: Age-period-cohort
HPV: Human papillomavirus
